# Clinical Characteristics and Outcomes of Patients With Antithyroid Drug–Related Liver Injury

**DOI:** 10.1210/jendso/bvad133

**Published:** 2023-10-27

**Authors:** Adeel Ahmad Khan, Fateen Ata, Afia Aziz, Hana Elamin, Aamir Shahzad, Zohaib Yousaf, Anthony Donato

**Affiliations:** Department of Endocrinology, Hamad Medical Corporation, 3050, Doha, Qatar; Department of Endocrinology, Hamad Medical Corporation, 3050, Doha, Qatar; Department of Internal Medicine, Hamad Medical Corporation, 3050, Doha, Qatar; National University-Sudan, Khartoum 11115, Sudan; Department of Medicine, Tower Health, West Reading, PA 19611, USA; Department of Medicine, Tower Health, West Reading, PA 19611, USA; Department of Medicine, Tower Health, West Reading, PA 19611, USA

**Keywords:** antithyroid drugs, carbimazole, methimazole, propylthiouracil, liver injury, hepatotoxicity

## Abstract

**Context:**

Antithyroid drugs (ATDs) are the cornerstone of hyperthyroidism management. Hepatotoxicity due to ATDs can range from mild transaminase elevation to liver transplantation requirement and mortality.

**Objective:**

The primary objective of the systematic review was to assess the clinical characteristics and outcomes of patients with drug induced liver injury (DILI) due to ATDs.

**Methods:**

We conducted a systematic review of PUBMED, SCOPUS, and EMBASE on characteristics and outcomes of adults (>18 years) with DILI due to ATDs. We defined DILI as bilirubin ≥2.5 mg/dL or international normalized ratio >1.5 with any rise in alanine aminotransferase (ALT), aminotransferase (AST), or alkaline phosphatase (ALP), or an elevation of ALT or AST >5 times or ALP >2 times the upper limit of normal without jaundice/coagulopathy.

**Results:**

The review included 100 articles describing 271 patients; 148 (70.8%) were female (N = 209). Mean age was 42.9 ± 17.2 years. Graves’ disease was the most common indication for ATDs. Carbimazole/methimazole (CBM/MMI) was the most common offending agent (55.7%). DILI pattern was hepatocellular in 41.8%, cholestatic in 41.3%, and mixed in 16.9%. Outcomes included death in 11.8%, liver transplantation in 6.4%, partial improvement in 2.2%, and complete resolution in 79.6% with a median time (IQR) to resolution of 45 (20-90) days. Patients in the propylthiouracil (PTU) group had higher initial bilirubin, initial AST, initial ALT, peak ALT, peak AST, severe and fatal DILI, liver transplantation, and mortality than CBM/MMI. Rechallenge of antithyroid medication was infrequently reported (n = 16) but was successful in 75%.

**Conclusion:**

DILI due to ATDs can present with different patterns and should prompt immediate drug discontinuation. Referral to a hepatologist should be considered if severe as transplantation is sometimes required. PTU-induced DILI may have worse outcomes than CBM/MMI.

Hyperthyroidism (HT) is a common clinical condition with a prevalence of up to 0.8% in Europe and 0.5% in the United States [1]. Antithyroid drugs (ATDs), which include methimazole (MMI), carbimazole (CBM), and propylthiouracil (PTU), are the cornerstone of HT management. Long-term ATD use is 1 of the recommended definitive treatment options in patients with HT, especially due to Graves disease [2]. However, ATDs have several side effects. Minor side effects, which can occur in up to 15% of cases, include skin rashes, itching, joint pain, swelling, nausea, and vomiting. These do not usually require drug discontinuation and can be managed with antihistamines or reduction of ATD dosage. Major side effects of ATDs can occur in up to 1% of patients, including neutropenia, vasculitis, aplastic anemia, and hepatitis. Rare side effects such as pancreatitis and hypoglycemia due to the development of anti-insulin antibodies have also been reported [3]. These major side effects necessitate immediate discontinuation and possible hospitalization for management.

Hepatotoxicity is one of the most common and severe side effects of ATDs. The prevalence of ATD-related liver injury is variable depending on the type of ATD. Suzuki et al reported an overall prevalence of ATD-related drug-induced liver injury (DILI) at 2.5%, with 1.4% due to MMI and 6.3% due to PTU [4]. The severity of DILI due to ATDs varies, ranging from asymptomatic transaminase elevation to symptomatic hepatitis to the requirement for liver transplantation and mortality [5, 6]. PTU is mostly attributed to more severe forms of DILI than CBM/MMI [4]. However, reports of CBM/MMI-related acute liver failure have also been reported in the literature [7]. Moreover, ATDs can lead to different patterns of liver injury [5, 8]. Cholestatic and hepatocellular liver injuries have been reported with both MMI/CBM and PTU [9-11].

The most effective treatment for ATD-related DILI is discontinuation of the drug [12]. However, due to the different hepatotoxicity profiles of PTU and CBM/MMI, several cases of the retrial of the alternate ATD have been reported with variable outcomes. Becker et al reported successful treatment of Graves disease with PTU after the MMI discontinuation due to hepatotoxicity development [13]. On the other hand, Livadas et al report progressive liver biochemistry worsening following switching from MMI to PTU [14]. Most of the data on the hepatotoxicity profile, management, and outcomes of patients with ATD-induced liver injury is based on observational studies and case reports. We conducted a systematic review on patients with ATD-induced hepatotoxicity to provide comprehensive and robust evidence on clinical characteristics and outcomes of patients with these widely used drugs.

## Materials and Methods

### Literature Search

We performed a literature search using PUBMED, SCOPUS, and EMBASE to identify eligible articles published in English from any date until December 31, 2022. The study design and methods were carried out according to the PRISMA guidelines [15]. The search strategy consisted of terms and synonyms for antithyroid medications, including “carbimazole,” “methimazole,” “propylthiouracil,” “antithyroid,” “thyroid blocking,” “thyroid antagonist.” These were combined with terms for liver disease including “jaundice,” “liver injury,” “liver disease,” “liver disorder,” “liver dysfunction,” “hepatitis,” “hepatotoxic,” “cholestatic,” “cholestasis,” “biliary stasis,” and “hepatocellular,” “mixed,” combined using the Boolean operator “OR.”

### Study Selection

Three members of the study team (A.A.K., F.A., A.D.) independently screened the titles and abstracts of the retrieved articles using RAYYAN AI software [16], followed by full-text screening of included articles. Each article was screened by 2 reviewers. In case of disagreement between the 2 reviewers, a fourth reviewer (Z.Y.) independently reviewed to make a final decision regarding the inclusion or exclusion of the article.

### Inclusion Criteria

Studies (case reports, case series, letter to editor, retrospective studies, prospective studies, and randomized controlled trials) in English reporting data on DILI due to ATDs in adult patients (age 18 years and above) were included in the systematic review. We defined DILI as per the criteria described by Chalasani et al. [17], which included the fulfillment of either of the following: (1) Presence of jaundice (serum bilirubin ≥2.5 mg/dL) or hypocoagulability (international normalized ratio >1.5) with any rise in alanine aminotransferase (ALT) or aspartate aminotransferase (AST), or alkaline phosphatase (ALP) levels, or (2) in the absence of jaundice or coagulopathy, elevations of ALT or AST above 5 times, or ALP above 2 times the upper limit of normal.

### Exclusion Criteria

Studies not fulfilling the DILI criteria mentioned above, those in languages other than English, conference abstracts, review articles, and studies on pediatric patients (defined as age <18) were excluded.

### Assessment of Causality of DILI

To assess the likelihood of causality of DILI due to ATDs described in the included article, we calculated the Roussel Uclaf Causality Assessment Method (RUCAM) score wherever possible. Based on the RUCAM score, the likelihood of DILI due to ATDs was adjudged to be as follows: 0 or less than 0 as “excluded,” 1 to 2 as “unlikely,” 3 to 5 as “possible,” 6 to 8 as “probable,” and more than 8 as “highly probable” [18].

### Assessment of the Pattern of DILI

The clinical pattern of liver injury was assessed by calculating the R factor using the ALT and ALP values at the initial presentation. An R-value of <2 indicates a cholestatic pattern, 2 to 5 mixed pattern, and >5 denotes a hepatocellular pattern of liver injury [17].

### Assessment of Severity of DILI

DILI was categorized into 5 groups of severity using the criteria described by Chalasani et al [17]. Patients with any degree of serum liver enzyme elevation in the absence of jaundice (bilirubin <2.5 mg/dL) were categorized as “mild,” those with jaundice (bilirubin >2.5 mg/dL) or hypocoagulability (INR > 1.5) without the requirement for hospital admission were labeled as “moderate,” patients with jaundice and/or hypocoagulability requiring hospitalization were grouped into “moderately severe,” those characterized by jaundice and liver failure or any other organ failure as “severe,” and the ones with death due to liver disease or liver transplantation within 6 months of DILI were categorized as “fatal” [17].

### Quality Assessment

Two authors (A.A. and H.E.) independently assessed the quality of the included studies. We used the Joanna Briggs Institute case report appraisal checklist for inclusion in systematic reviews to assess case reports/case series, whereas the Methodological Index for Non-randomized Studies scoring system was used to assess observational studies [19, 20]. A.A.K. conducted an independent assessment to resolve the conflict in case of disagreement between the 2 reviewers.

### Data Collection

A.A.K., A.A., H.E., Z.Y., and A.S. collected data into a predesigned data collection sheet for the included studies. In case of a lack of availability of individualized patient data, the corresponding authors of the studies were contacted by A.A.K. to request the data. The collected variables included demographic characteristics, including patient age, gender, comorbid conditions, cause of HT, type, duration and dosage of ATD. Extracted laboratory data included initial and peak serum bilirubin (mg/dL), AST (U/L), ALT (U/L), and ALP (U/L). Data on the serology of hepatitis A virus, hepatitis B virus, hepatitis C virus, cytomegalovirus, Epstein–Barr virus, herpes simplex virus, HIV, and autoimmune profiles were also collected whenever reported. Data on treatment included information on the continuation/discontinuation of ATDs, the retrial of alternate ATDs, the outcome of the retrial (if applicable), and the use of N-acetylcysteine and steroids. Data on outcomes included resolution of liver injury (defined by complete normalization of bilirubin/liver enzymes), improvement (defined by reporting of decrease in bilirubin/liver enzymes but lack of reporting of complete resolution), liver transplantation requirement, and mortality/death due to ATDs.

### Statistical Analysis

We described continuous variables using means ± SD or median with interquartile range (IQR), and comparisons were reported using independent t-test, Mann–Whitney U test, 1-way analysis of variance or Kruskal–Wallis as appropriate. Categorical variables were reported as total numbers and percentages, and the groups were compared using the chi-square test or Fisher's exact test. *P* < .05 was considered to be statistically significant. STATA 17 (STATA, LLC, College Station, TX) was used for the statistical analysis.

### Protocol Registration

The protocol for the systematic was registered with the International Prospective Register of Systematic Reviews (PROSPERO) with the protocol ID CRD42023388961.

## Results

### Search Results

**Figure 1.**
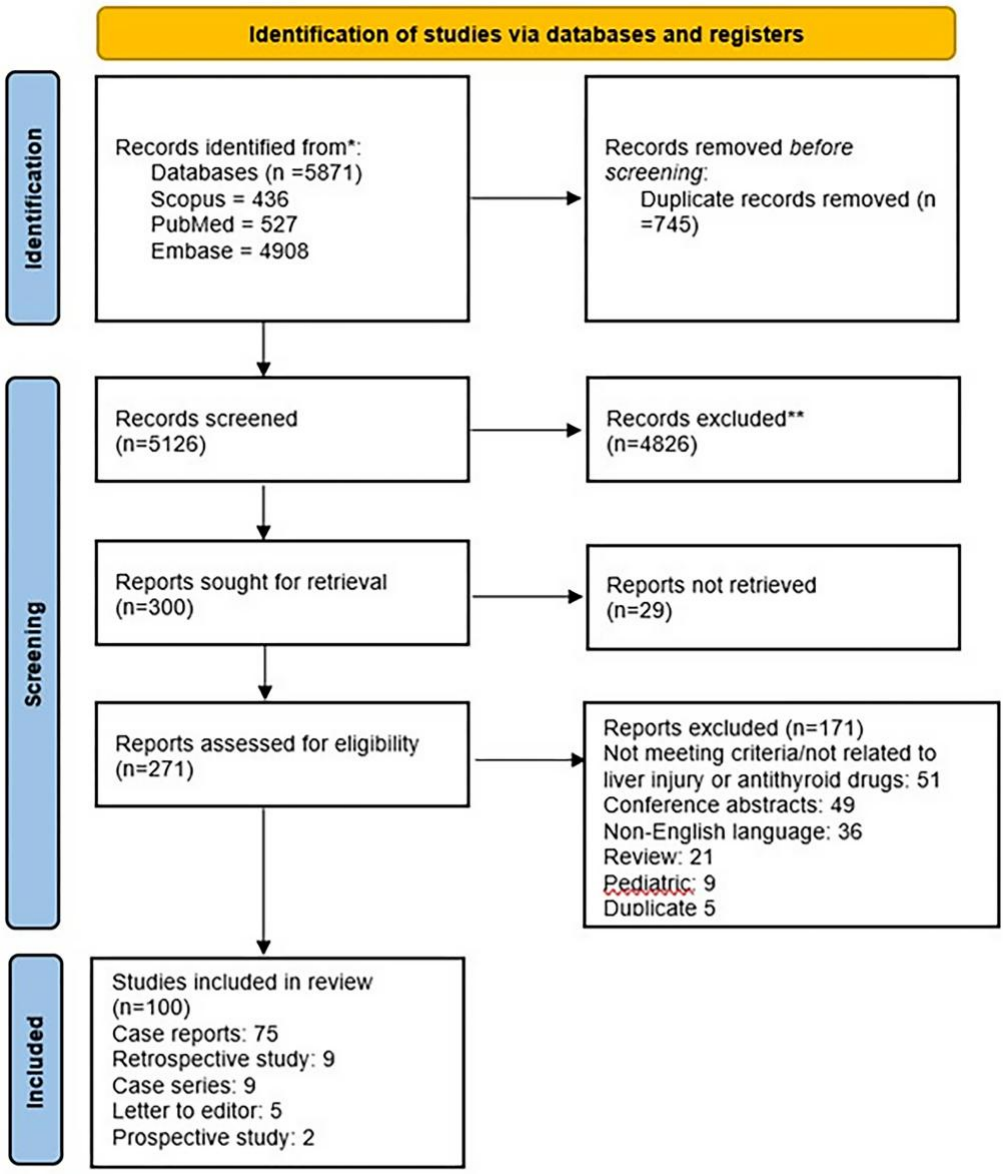
PRISMA flowsheet of the article screening process.

A total of 300 relevant articles were identified after the initial screening. Following the full-text review, 200 articles were excluded. The final systematic review included 100 articles comprising 75 case reports, 9 case series, 5 letters to editor, 9 retrospective, and 2 prospective studies (Fig. 1). Two hundred and seventy-one patients who developed DILI due to ATDs were identified from the 100 included studies. Individual patient data were available for 100 patients from 90 studies (75 case reports, 9 case series, 5 letters to editor, and 1 retrospective study). Table 1 summarizes the data from 11 studies included in the systematic review.

**Table 1.** Summary of studies included in the systematic review

Authors	Number of patients	Gender	Type of ATD	Type of liver injury
Aithal and Day (1999) [21]	1	F = 1	PTU = 1	N/A
Anderson et al (2016) [22]	10	M = 4 F = 6	MMI = 7 PTU = 3	N/A
Kim et al (2001) [23]	6	M = 3 F = 3	PTU = 1 N/A = 5	N/A
Huang et al (2022) [24]	34	N/A	CBM = 13 PTU = 21	N/A
Nash et al (2021) [25]	1	N/A	CBM = 1	N/A
Dag et al (2014) [26]	2	M = 1 F = 1	N/A	Hepatocellular = 1 Cholestatic = 1
Li et al (2007) [27]	23	N/A	N/A	Hepatocellular = 12 Cholestatic = 11
Takata (1977) [28]	1	F = 1	PTU = 1	Hepatocellular = 1
Wang et al (2009) [29]	2	F = 2	MMI = 1 PTU = 1	Hepatocellular = 1 Cholestatic = 1
Wang and Chen (2022) [30]	3	N/A	N/A	N/A
Yang et al (2015) [31]	90	M = 28 F = 62	MMI = 51 PTU = 39	Hepatocellular = 44 Cholestatic = 25 Mixed = 21

Abbreviations: ATD, antithyroid drug; CBM, carbimazole; F, females; M, males; MMI, methimazole; N/A, not available; PTU, propylthiouracil.

### Characteristics and Outcomes of Data From all the Included Articles

Table 2 summarizes the demographic, laboratory tests, and outcomes of 271 patients with ATD-related DILI included in the systematic review. Gender details were available for 209 patients, of which 148 (70.8%) patients were female, and 61 (29.2%) were male. The mean (SD) age was 42.9 ± 17.2 years. Graves disease (n = 152, 80%) was the most common diagnosis, followed by toxic multinodular goiter (n = 4, 2.1%), toxic adenoma (n = 2, 1%), and thyroiditis (n = 1, 0.53%). One hundred and thirty-two (55.7%) patients had DILI due to carbimazole/methimazole (CBM/MMI) and 105 (44.3%) due to PTU. The mean (SD) doses of CBM and MMI were 29.4 ± 10.9 and 30.5 ± 12.4 mg/day, respectively. The median dose of PTU was 300 (150-450) mg/day. A cholestatic pattern was present in 78 (41.3%), a hepatocellular pattern in 79 (41.8%), and 32 (16.9%) had a mixed pattern of liver injury. A total of 70 patients had sufficient data available to calculate the RUCAM score. Based on the RUCAM score, 5 (7.1%) patients had “highly probable,” 43 (61.4%) had “probable,” 21 (30%) had “possible,” and 1 (1.4%) had “unlikely” categorization of liver injury due to ATDs. The median (IQR) duration of ATD treatment prior to the onset of DILI was 30 (14-90) days.

**Table 2.** Baseline characteristics and outcomes of all patients with ATD-related DILI

Variable	Result
Number of patients, n	271
Gender, n (%)	n = 209
Female	148 (70.8)
Male	61 (29.2)
Age, years, mean ± SD (n = 101)	42.9 ± 17.2
Diagnosis, n (%)	n = 190
Graves disease	152 (80)
Toxic MNG	4 (2.1)
Thyroiditis	1 (0.53)
Toxic adenoma	2 (1)
Unspecified hyperthyroidism	31 (16.3)
Comorbid conditions	n = 100
CKD	1 (1)
CAD	2 (2)
Heart failure	3 (3)
Chronic hepatitis B virus	2 (2)
Type of ATD, n (%)	n = 237
Carbimazole/methimazole	132 (55.7%)
Propylthiouracil	105 (44.3%)
Dose of ATD (n = 91), mg/day	
Carbimazole, mean± SD	29.4 ± 10.9
Methimazole, mean± SD	30.5 ± 12.4
Propylthiouracil, median (IQR)	300 (150-450)
Type of liver injury, n (%)	n = 189
Cholestatic	78 (41.3)
Hepatocellular	79 (41.8)
Mixed	32 (16.9)
RUCAM score, n (%)	n = 70
1-2 (unlikely)	1 (1.4)
3-5 (possible)	21 (30)
6-8 (probable)	43 (61.4)
>8 (highly probable)	5 (7.1)
Duration of treatment prior to DILI, days, median (IQR) (n = 98)	30 (14-90)
First bilirubin, mg/dL, median (IQR) (n = 92)	12.5 (4.75-0.25)
Highest bilirubin, mg/dL, median (IQR) (n = 68)	21.4 (8.1-30.6)
First AST, U/L, median (IQR) (n = 86)	163 (69-540)
Highest AST, U/L, median (IQR) (n = 56)	361.5 (135-1199)
First ALT, U/L, median (IQR) (n = 86)	200 (94-852)
Highest ALT, U/L, median (IQR) (n = 56)	426.5 (137-1080)
First ALP, U/L, median (IQR) (n= 83)	289 (200-399)
Highest ALP, U/L, median (IQR) (N = 55)	353 (242–509)
Severity of DILI, n (%)	n = 100
Mild	11 (11)
Moderate	5 (5)
Moderately severe	59 (59)
Severe	11 (11)
Fatal	14 (14)
	n = 100
Autoimmune hepatitis, n (%)	4 (4)
ASMA	2 (2)
AMA	1 (1)
Management, n (%)	n = 100
N-acetylcysteine + steroids	2 (2)
Steroids only	35 (35)
Retrial of alternative ATD	16 (16)
Successful retrial	12 (75)
No improvement in liver function tests	1 (6.25)
Retrial caused DILI again	3 (18.75)
Thyroidectomy performed for the management of thyrotoxicosis, n (%)	N = 110 18 (16.4)
Outcomes of DILI, n (%)	n = 93
Complete resolution improvement	74 (79.6)
Liver transplantation	2 (2.2)
Mortality	6 (6.4)
	11 (11.8)
Time to resolution, days, median (IQR) (n = 65)	45 (2090)

Abbreviations: ALP, alkaline phosphatase; ALT, alanine aminotransferase; AMA, antimitochondrial antibody; ANA, antinuclear antibody; ASMA, antismooth muscle antibody; AST, aspartate aminotransferase; ATD, antithyroid drug; CAD, coronary artery disease; CKD, chronic kidney disease; DILI, drug-induced liver injury; MNG, multinodular goiter; RUCAM, Roussel Uclaf Causality Assessment Method.

At presentation, the median (IQR) bilirubin was 12.5 (4.75-20.25) mg/dL, AST was 163 (69-540) U/L, ALT was 200 (94-852) U/L, and ALP was 289 (200-399) U/L. The highest median (IQR) bilirubin was 21.4 (8.1-30.6) mg/dL, AST was 361.5 (135-1199) U/L, ALT was 426.5 (137-1080) U/L, and ALP was 353 (242-09) U/L. In terms of severity of DILI, the majority (n = 59; 59%) had “moderately severe,” followed by “severe” (n = 11, 11%), “fatal” (n = 14, (14%), “mild” (n = 11, 11%), and “moderate” (n = 5, 5%) DILI. Four patients had autoimmune hepatitis due to DILI.

For the management of patients, steroids were used in 35 (35%) patients, while a combination of N-acetylcysteine plus steroids was used in 2 (2%) patients. After the development of DILI on ATD, 18 (16.4%) patients required thyroidectomy to manage HT. Outcomes of DILI were reported in 93 patients, of which 74 (79.6%) had complete resolution, and 2 (2.2%) had improvement in the liver function tests. Six (6.4%) patients underwent liver transplantation, whereas 11 (11.8%) died. In patients who had a resolution of DILI, the median (IQR) time to resolution was 45 (20-90) days.

### Comparison in Clinical Characteristics and Outcomes based on the Type of ATD

Table 3 describes the differences in patients treated with CBM/MMI and PTU for the studies for which individual patient data were available. There was a female predominance in the PTU group compared with the CBM/MMI group (89.7% vs 64.4%, *P* = .004). Patients in the CBM/MMI group were older than in the PTU group (mean ± SD of 48.8 ± 2.2 vs 33.2 ± 2 years, *P* < .001). The cholestatic pattern of liver injury was more common in CBM/MMI group (67.4% vs 41.4%), the hepatocellular pattern was more common in the PTU group (28.6% vs 13.9%), and the mixed pattern was seen more in CBM/MMI group (18.6% vs 10.3%). All these differences were statistically significant (*P* = .008). Patients in the PTU group had a more prolonged duration of treatment prior to the onset of DILI (median [IQR] of 75 [21-128] vs 28 [14-42] days, *P* = .002). PTU patients also had higher initial bilirubin (median [IQR] of 16.2 [5.9-22.4] vs 8.42 [3.8-18] mg/dL, *P* = .04), initial AST (median [IQR] of 565.5 [138-1127] vs 94 [43-209] U/L, < 0.001), and initial ALT (median [IQR] of 918 [238-1227] vs 135 [86-251] U/L, *P* < .001) at DILI onset. Peak levels of ALT (median [IQR] of 1002.5 [421-1442] vs 215 [114-755] U/L, *P* = .001), and AST (median [IQR] of 866 [276-1693] vs 187 [105-605] U/L, *P* = .001) were also higher in the PTU group than in the CBM/MMI group. The PTU cohort also had more patients who developed severe (17.9% vs 6.7%, *P* = .001) and fatal (28.2% vs 5%, *P* = .001) liver injuries. Resolution of DILI was reported more in CBM/MMI group than the PTU group (90.7% vs 68.6%, 0.003). However, patients in the PTU group underwent more liver transplantation (17.1% vs 0%, *P* = .003) and more died (14.3% vs 5.6%, *P* = .003) than the CBM/MMI group. No statistically significant difference in time taken for the resolution of DILI was noted between the 2 groups.

**Table 3.** Comparison of clinical characteristics and outcomes of patients with DILI based on type of ATD

Variable	Carbimazole/Methimazole (60)	Propylthiouracil (39)	*P*-value
Gender, n (%)	n = 59	n = 39	
Female	38 (64.4)	35 (89.7)	.004
Male	21 (35.6)	4 (10.3)	
Age, years, mean ± SD (n = 98)	48.8 ± 2.2	33.2 ± 2	<.001
Diagnosis, n (%)	n = 60	n = 39	
Graves disease	34 (56.7)	28 (71.8)	.1
Toxic MNG	1 (1.66)	3 (7.7)	
Thyroiditis	1 (1.66)	0	
Toxic adenoma	2 (3.33)	0	
Unspecified	22 (36.66)	8 (20.5)	
Type of liver injury, n (%)	n = 43	n = 29	
Cholestatic	29 (67.4)	12 (41.4)	.008
Hepatocellular	6 (13.9)	14 (28.6)	
Mixed	8 (18.6)	3 (10.3)	
Duration of treatment prior to DILI, days, median (IQR) (n = 97)	28 (14-42)	75 (21-128)	.002
First bilirubin, mg/dL, median (IQR) (n = 91)	8.42 (3.8-18)	16.2 (5.9-22.4)	.04
Highest bilirubin, mg/dL, median (IQR) (n = 67)	19.84 (5.3-30.1)	22.8 (15.9-29.5)	.4
First AST, U/L, median (IQR) (n = 84)	94 (43-209)	565.5 (138-1127)	<.001
Highest AST, U/L, median (IQR) (n = 55)	187 (105-605)	866 (276-1693)	.001
First ALT, U/L, median (IQR) (n = 84)	135 (86-251)	918 (238-1227)	<.001
Highest ALT, U/L, median (IQR) (n = 55)	215 (114-755)	1002.5 (421-1442)	.001
First ALP, U/L, median (IQR) (n = 83)	328 (207-440)	266 (186-364)	.15
Highest ALP, U/L, median (IQR) (n = 55)	364 (301-556)	293.5 (217-445)	.2
Severity of DILI, n (%)	n = 60	n = 39	
Mild	9 (15)	2 (5.1)	.001
Moderate	5 (8.33)	0	
Moderately severe	39 (65)	19 (48.8)	
Severe	4 (6.7)	7 (17.9)	
Fatal	3 (5)	11 (28.2)	
Overall outcome of DILI, n (%)	n = 54	n = 35	
Resolution	49 (90.7)	24 (68.6)	.003
Improved	2 (3.7)	0	
Liver transplantation	0	6 (17.14)	
Mortality	3 (5.55)	5 (14.3)	
Time to resolution of DILI, days (n = 65)	57.5 (15-84)	40 (28-160)	.25

Abbreviations: ALP, alkaline phosphatase; ALT, alanine aminotransferase; AST, aspartate aminotransferase; ATD, antithyroid drug; DILI, drug-induced liver injury; MNG, multinodular goiter.

### Comparison in Clinical Characteristics and Outcomes based on Resolution/Nonresolution of DILI

Table 4 summarizes the differences in clinical characteristics and outcomes of patients. Seventy-six reports described the resolution of liver injury (complete resolution = 74, improvement reported = 2), while 14 did not resolve (liver transplantation = 6, mortality = 8). Patients with resolution of DILI were older (mean age of 43.4 ± 17.6 vs 32.2 ± 10.6 years, *P* = .02) and had a shorter duration of treatment with ATD prior to the onset of liver injury (median [IQR] of 30 [14-60] vs 128 [60-180] days, *P* = .001). Moreover, patients not having a resolution of DILI had a higher initial bilirubin (median [IQR] of 20.8 [14.6-29.5] vs 8.96 [3.8-19] mg/dL, *P* = .001), a higher initial AST (median [IQR] of 767 [135-948] vs 147 [67-384] U/L, *P* = .01), higher initial ALT (median [IQR] of 674 [180-1000] vs 169.5 [89.5 vs 567.5] U/L, *P* = .01), higher peak bilirubin (median [IQR] of 29.5 [22-50] vs 20.8 [7.7-26] mg/dL, *P* = .02), and a higher peak ALT (median [IQR] of 947.5 [390.5-1163.5] vs 323 [123-942] U/L, *P* = .04) than patients who had resolution of liver injury. There were no differences in gender and diagnosis of HT between the 2 groups.

**Table 4.** Comparison of clinical characteristics and outcomes of patients with DILI who had resolution of DILI to those without resolution

Variable	Resolution (76)	No resolution (14)	*P* value
Gender, n (%)	n = 75	n = 14	
Female	54 (72)	11 (78.6)	.6
Male	21 (28)	3 (21.4)	
Age, years, mean (SD) (n = 89)	43.4 ± 17.6	32.2 ± 10.6	.02
Diagnosis, n (%)	n = 76	n = 14	.6
Graves disease	49 (64.5)	8 (57.1)	
Toxic MNG	4 (5.3)	0	
Thyroiditis	1 (1.3)	0	
Toxic adenoma	2 (2.6)	0	
Unspecified	20 (26.3)	6 (42.9)	
Type of liver injury, n (%)	n = 61	n = 10	
Cholestatic	36 (59)	4 (40)	.25
Hepatocellular	15 (24.6)	5 (50)	
Mixed	10 (16.4)	1 (10)	
Duration of treatment prior to DILI, days, median (IQR) (n = 88)	30 (14-60)	128 (60-180)	.001
First bilirubin, mg/dL, median (IQR) (n = 84)	8.96 (3.8-19)	20.8 (14.6-29.5)	.001
Highest bilirubin, mg/dL, median (IQR) (n = 63)	20.8 (7.7-26)	29.5 (22-50)	.02
First AST, U/L, median (IQR) (n = 79)	147 (67-384)	767 (135-948)	.01
Highest AST, U/L, median (IQR) (n = 51)	276 (134-1260)	924 (320.5-1265.5)	.15
First ALT, U/L, median (IQR) (n = 81)	169.5 (89.5-567.5)	674 (180-1000)	.01
Highest ALT, U/L, median (IQR) (n = 53)	323 (123-942)	947.5 (390.5-1163.5)	.04
First ALP, U/L, median (IQR) (n = 80)	303 (200-401)	217 (170-364)	.3
Highest ALP, U/L, median (IQR) (n = 52)	368 (289-520)	279.5 (217-509)	.36
Severity of DILI, n (%)	n = 76	n = 14	
Mild	10 (13.2)	0	<.001
Moderate	5 (6.6)	0	
Moderately severe	52 (68.4)	0	
Severe	9 (11.8)	0	
Fatal	0	14 (100)	

Abbreviations: ALP, alkaline phosphatase; ALT, alanine aminotransferase; AST, aspartate aminotransferase; ATD, antithyroid drug; DILI, drug-induced liver injury; MNG, multinodular goiter.

### Comparison in Clinical Characteristics and Outcomes based on Patterns of DILI

There were no statistically significant differences between different patterns of DILI (cholestatic, hepatocellular and mixed) in terms of gender, age, diagnosis of HT, initial and peak bilirubin levels, and severity and outcomes of DILI (Table 5).

**Table 5.** Comparison of clinical characteristics and outcomes of patients with different patterns of DILI

Variable	Cholestatic (41)	Hepatocellular (20)	Mixed (11)	*P*-value
Gender, n (%)	n = 40	n = 20	n = 11	
Female	25 (62.5)	18 (90)	8 (72.7)	.08
Male	15 (37.5)	2 (10)	3 (27.3)	
Age, years, mean ± SD (n = 71)	43.8 ± 17.6	36.7 ± 11.2	40.3 ± 17.8	.09
Diagnosis, n (%)	n = 41	n = 20	n = 11	
Graves disease	23 (56.1)	14 (70)	9 (81.8)	.2
Toxic MNG	1 (2.4)	0	1 (9.1)	
Thyroiditis	1 (2.4)	0	0	
Toxic adenoma	0	1 (5)	0	
Unspecified	16 (39)	5 (25)	1 (9.1)	
Duration of treatment prior to DILI, days, median (IQR) (n = 71)	30 (19-60)	90 (30-120)	28 (14-60)	.07
First bilirubin, mg/dL, median (IQR) (n = 67)	12.1 (4.4-16.7)	19 (6.4-22.8)	6.2 (4.5-29.5)	.3
Highest bilirubin, mg/dL, median (IQR) (n = 50)	20.4 (7.7-25.9)	23.1 (19-46.8)	25.2 (13.4-31.7)	.2
Severity of DILI, n (%)	n = 41	n = 20	n = 11	
Mild	5 (12.2)	4 (20)	1	.28
Moderate	4 (9.7)	0	0	
Moderately severe	24 (58.5)	7 (35)	8	
Severe	4 (9.7)	4 (20)	1	
Fatal	4 (9.8)	5 (25)	1	
Overall outcome, n (%)	n = 40	n = 20	n = 11	
Resolution	35 (87.5)	15 (75)	10 (90.9)	.5
Improved	1 (2.5)	0	0	
Liver transplantation	1 (2.5)	3 (12)	0	
Mortality	3 (7.5)	2 (10)	1 (9.1)	
Time to resolution (n = 53)	60 (28-90)	40 (30-160)	15 (10-42)	.09

Abbreviations: ATD, antithyroid drug; DILI, drug-induced liver injury; MNG, multinodular goiter.

### Outcomes of Retrial of Alternate ATD

Table 6 summarizes the characteristics of DILI patients who underwent a retrial of an alternate ATD. After discontinuation of ATD causing the DILI, a trial of the alternate ATD was used in 16 (16%) patients, of whom 10 (62.5%) were on CBM/MMI, and 6 (37.5%) were on PTU. 12 (75%) of these patients had a successful retrial without the further development of DILI. Seven (70%) patients on CBM/MMI and 5 (83.3%) on PTU had a successful retrial. The initial liver injury in the majority of these patients was moderately severe (n = 9, 25%), followed by mild (n = 2, 16.7%) and moderate (n = 1, 8.3%) DILI.

**Table 6.** Characteristics and outcomes of patients with a retrial of alternate ATD

Variable	Results
Number of patients	16
Type of ATD, n (%)	n = 16
CBM/MMI	10 (62.5)
PTU	6 (37.5)
Successful retrial, n (%)	n = 12
CBM/MMI	7 (70)
PTU	5 (83.3)
Severity of DILI, n (%)	n = 16
Mild	2 (12.5)
Moderate	2 (12.5)
Moderately severe	11 (68.75)
Fatal	1 (6.25)
Initial severity of DILI in patients with successful retrial, n (%)	n = 12
Mild	2 (16.7)
Moderate	1 (8.3)
Moderately severe	9 (75)

Abbreviations: ATD, antithyroid drugs; CBM, carbimazole; DILI, drug-induced liver injury; MMI, methimazole; PTU, propylthiouracil.

## Discussion

In this systematic review assessing DILI due to ATDs, cholestatic and hepatocellular patterns of the liver had similar prevalence. The majority had “moderately severe” liver injury, although this finding likely involved publication bias, as milder cases may be less likely to be represented in case reports. The majority had a complete resolution of DILI. The cholestatic pattern and mixed patterns of liver injury were more common in the CBM/MMI group, while the hepatocellular pattern was more common in the PTU group. Patients using PTU had higher initial bilirubin, initial AST, initial ALT peak ALT, peak AST, severe and fatal DILI, liver transplantation, and mortality than CBM/MMI patients. After discontinuation of ATD causing the DILI, a trial of the alternate ATD was successful in 75% (12 out of 16) patients.

ATDs have been used to treat HT for more than 8 decades [32]. PTU was the favored ATD before the 1990s [33, 34]. However, in recent times, the use of MMI/CBM has increased. PTU constituted around two-thirds of ATD prescriptions prior to 1995. However, following 1996, the prescriptions for MMI took over. In 2008, PTU only constituted 22% of the total new ATD prescriptions [33]. This is probably related to the increased safety profile and availability of once-daily dosing frequency of CBM/MMI compared with the PTU. Studies comparing the side-effect profile of ATDs show an increased risk of liver injury with PTU compared with CBM/MMI. Nakamura et al reported a 29.6% increased risk of elevation in transaminases twice the upper limit of normal compared with only 6.6% with MMI use [35]. Yu et al also reported a higher risk of DILI with PTU compared to CBM/MMI use, with an odds ratio of 2.40 (*P* = .02) [36]. Furthermore, PTU use is also more associated with the development of more severe forms of DILI than other ATDs [4, 6], similar to our findings.

The duration of onset of hepatotoxicity after ATD initiation is rarely studied. Our review revealed a longer duration of ATD treatment prior to DILI development in the PTU group than the CBM/MMI group. Our results are consistent with those reported by Otsuka et al, who found that patients on PTU developed hepatotoxicity later than the MMI group [37]. Similarly, Yu et al also found similar results with DILI due to CBM/MMI occurring earlier than PTU [36]. Knowledge of the differences in the duration of onset of DILI is of particular importance to clinicians as it can alert them of the possibility of an underlying DILI even if the drug was initiated much earlier before the development of liver injury.

PTU and CBM/MMI are associated with different patterns of liver injury [5, 8]. Findings associated with hepatocellular injury in PTU include parenchymal necrosis and hemorrhage, lobular structure disruption, and mixed inflammatory infiltration. On the other hand, portal tract inflammation, intracanalicular cholestasis, and microvascular steatosis have been noted in MMI-related cholestatic injuries [38]. Due to these differential hepatotoxicity profiles, a trial of alternate ATD can be considered if 1 ATD leads to hepatoxicity. However, careful selection of patients is required prior to the trial of alternate ATD because although the cholestatic liver injury is more common with CBM/MMI and hepatocellular with PTU, cases of CBM/MMI related hepatocellular DILI and PTU-related cholestatic DILI have been reported as well [9, 31, 39]. In our review, patients who underwent a successful retrial of the alternate ATD had mild to moderately severe DILI at the onset. A trial of alternate ATD in patients with a less severe liver injury can alleviate the need for performing emergency definitive therapies for HT, such as thyroidectomy, and prevent the development of complications of uncontrolled HT like atrial fibrillation and thyroid storm.

Another significant finding in our review is the lower age in patients with PTU-related DILI compared with CBM/MMI. Our results are similar to the ones reported by Suzuki et al, who found PTU-induced DILI in the younger age group [4]. Huang and Liaw also reported patients <30 years old to be at the highest risk of PTU-induced liver injury [40]. The exact mechanism of this association remains unclear. However, a higher prevalence of other risk factors for liver injury, like alcohol intake in younger age groups, could potentially contribute to the increased risk of liver injury, as could a more vigorous autoimmune response to the drug that might be seen in women of childbearing age [41].

### Strengths and Limitations

Our systematic review has several strengths. We used robust inclusion criteria based on a clear definition of liver injury, strengthening the review's methodology. Moreover, the RUCAM score was calculated to assess the causality of DILI and the R-factor to assess the DILI pattern wherever adequate information was available. However, since this systematic review only included patients who had already developed ATD-induced DILI, the prevalence of DILI due to ATDs could not be calculated. Publication bias likely limits our understanding of the spectrum of severity of disease, as it is quite likely that milder cases of DILI never made it to publication, as well as cases with a less than clear etiology as they may not have been suitable to publish. Hence, while our work shines a light on classic cases, it may not encompass the entire breadth of this problem nor its outcomes. Nevertheless, the review provides an essential insight into the different aspects of ATD-related DILI by combining evidence on features previously only available in small case reports and observational studies. Currently, there are no clear guidelines for screening DILI after ATD initiation. Further prospective studies are needed to make clear recommendations regarding routine screening of liver function tests after ATD initiation.

## Conclusion

CBM/MMI predominantly causes cholestatic pattern DILI, whereas PTU more frequently causes hepatocellular liver injury. Immediate discontinuation of ATD should take place upon recognition of liver injury. There are no current recommendations for prospective laboratory monitoring for liver injury in adults on ATDs. An early referral to a hepatologist should be considered in patients with severe DILI, especially in cases of PTU-induced hepatotoxicity due to the risk of requiring liver transplantation.

## Data Availability

Some or all datasets generated during and/or analyzed during the current study are not publicly available but are available from the corresponding author on reasonable request.

## Abbreviations

ALT: alanine aminotransferase
ALP: alkaline phosphatase
AST: aspartate aminotransferase
ATD: antithyroid drug
CBM: carbimazole
DILI: drug-induced liver injury
HT: hyperthyroidism
MMI: methimazole
PTU: propylthiouracil
RUCAM: Roussel Uclaf Causality Assessment Method
